# Shining a new light on the classical concepts of carbon‐isotope dendrochronology

**DOI:** 10.1111/nph.20258

**Published:** 2024-11-19

**Authors:** Thomas Wieloch

**Affiliations:** ^1^ Department of Forest Genetics and Plant Physiology Swedish University of Agricultural Sciences, Umeå Plant Science Centre 90183 Umeå Sweden; ^2^ Division of Geological and Planetary Sciences California Institute of Technology 91125 Pasadena CA USA

**Keywords:** carbon stable isotopes, dendrochronology, intramolecular isotope analysis, paleoclimate reconstruction, plant carbon fluxes, tree rings, water‐use efficiency, whole‐molecule isotope analysis

## Abstract

Retrospective information about plant ecophysiology and the climate system are key inputs in Earth system and vegetation models. Dendrochronology provides such information with large spatiotemporal coverage, and carbon‐isotope analysis across tree‐ring series is among the most advanced dendrochronological tools. For the past 70 years, this analysis was performed on whole molecules and, to this day, ^13^C discrimination during carbon assimilation is invoked to explain isotope variation and associated climate signals. However, recently it was reported that tree‐ring glucose exhibits multiple isotope signals at the intramolecular level (see Wieloch *et al*., 2025). Here, I estimated the signals' contribution to whole‐molecule isotope variation and found that downstream processes in leaf and stem metabolism each introduce more variation than carbon assimilation. Moreover, downstream processes introduce most of the climate information. These findings are inconsistent with the classical concepts/practices of carbon‐isotope dendrochronology. More importantly, intramolecular tree‐ring isotope analysis promises novel insights into forest metabolism and the climate of the past.

## Introduction

Tree rings are natural archives containing encoded information about plant metabolic processes, their environmental dependences, and the climate of the past. This information is (to a large extent) inaccessible to manipulation and monitoring experiments, and dendrochronologists strive to decipher it to contribute to a better understanding of the climate system, plant functioning, and biogeochemical cycles. Stable carbon‐isotope (^12^C, ^13^C) analysis across tree‐ring series is among the most advanced dendrochronological tools available today. This tool has (*inter alia*) been used to reconstruct leaf intrinsic water‐use efficiency (CO_2_ uptake relative to H_2_O loss, *iWUE*), air temperature, solar radiation, relative humidity, precipitation, and drought over past centuries at numerous locations world‐wide (Cernusak & Ubierna, 2022; Gagen *et al*., 2022).

Seventy years ago, tree‐ring ^13^C : ^12^C ratios were measured for the first time (Craig, 1953, 1954). While early studies analysed whole‐wood samples, most recent studies analyse cellulose, a glucose polymer extracted from tree rings to preclude error due to variation in wood composition (Helle *et al*., 2022). Note, arguments given below apply to glucose and cellulose but not necessarily to wood. Tree‐ring cellulose ^13^C : ^12^C data are commonly expressed in terms of ^13^C discrimination, *Δ*_trc_, denoting carbon‐isotope changes caused by physiological processes (Farquhar & Richards, 1984). Current data interpretation invokes a simplified mechanistic model of ^13^C discrimination accounting for two processes: CO_2_ diffusion from ambient air into leaf intercellular air spaces, and carbon assimilation by rubisco (Farquhar *et al*., 1982; McCarroll & Loader, 2004; Cernusak & Ubierna, 2022), combinedly termed diffusion‐rubisco (DR) discrimination (Wieloch *et al*., 2018).

Variation in DR discrimination depends on the ratio of intercellular‐to‐ambient CO_2_ concentration (Farquhar *et al*., 1982; Evans *et al*., 1986; Voelker *et al*., 2016). Intercellular CO_2_ concentration, in turn, varies with the rate of CO_2_ supply through leaf stomata and the rate of CO_2_ assimilatory demand. As stomata respond to moisture conditions, *Δ*_trc_ correlations with humidity parameters are generally assumed to derive from CO_2_‐supply‐side effects on DR discrimination (Gagen *et al*., 2022). By contrast, CO_2_ assimilation responds to temperature and solar radiation, and corresponding *Δ*_trc_ correlations are generally assumed to derive from CO_2_‐demand‐side effects on DR discrimination (Gagen *et al*., 2022). Moreover, there is a mechanistic relationship between DR discrimination and *iWUE* (Farquhar *et al*., 1982; Farquhar & Richards, 1984) which forms the basis of *iWUE* reconstructions from *Δ*_trc_ (Cernusak & Ubierna, 2022; Saurer & Voelker, 2022). *Nota bene*, all current *Δ*_trc_ interpretations assume DR discrimination governs *Δ*_trc_ variation (Gagen *et al*., 2022). Discrimination downstream of rubisco, denoted post‐rubisco (PR) discrimination (Wieloch *et al*., 2018), is considered constant for any given species (Gessler *et al*., 2014; Cernusak & Ubierna, 2022).

Recently, nuclear magnetic resonance spectroscopy was used (for the first time in dendrochronology) to measure intramolecular ^13^C discrimination, *Δ*_*i*_′, in glucose extracted across a series of tree rings from *Pinus nigra* Arnold (*i* denotes glucose carbon position C‐1 to C‐6; Supporting Information Notes S1) (Wieloch *et al*., 2018). Data of *Δ*_1_′, *Δ*_2_′, and *Δ*_3_′ pertaining to 1961–1980 (early period) and 1983–1995 (late period) were analysed separately as these series exhibit a change point in 1980 (Wieloch *et al*., 2025, pp. 1000–1017, in this issue of *New Phytologist*). Proposedly, the trees had access to groundwater during the early but not the late period (Wieloch *et al*., 2022a) causing metabolism affecting *Δ*_1_′ to *Δ*_3_′ to move from a homeostatic to a climate‐responsive state (Wieloch *et al*., 2025). By contrast, no change point was detected in *Δ*_4_′, *Δ*_5_′, and *Δ*_6_′. Based (*inter alia*) on multiple regression modelling, the dataset contains several ^13^C signals (Tables 1, S1; Fig. S1). First, vapour pressure deficit (*VPD*) affects both *Δ*_1_′ and *Δ*_3_′ during the late period (Wieloch *et al*., 2025). This relationship is thought to derive from DR discrimination. Additional leaf‐level ^13^C discrimination by phosphoglucose isomerase and/or glucose‐6‐phosphate dehydrogenase is thought to account for the stronger effect of *VPD* on *Δ*_1_′ compared to *Δ*_3_′. Second, during the late period, *Δ*_1_′ and *Δ*_2_′ are related to *ε*_met_ denoting hydrogen isotope fractionation by metabolic processes at glucose H^1^ and H^2^, and *ε*_met_ can be substituted by precipitation (*PRE*) without losing much of the models′ explanatory power (Wieloch *et al*., 2022a, 2025). These relationships are thought to derive from ^13^C discrimination by phosphoglucose isomerase and glucose‐6‐phosphate dehydrogenase in tree stems (Wieloch *et al*., 2025). Note, the described *Δ*_1_′ to *Δ*_3_′ models do not work for the early period (Wieloch *et al*., 2025). Third, global radiation (*RAD*) and temperature (*TMP*) affect *Δ*_4_′ to *Δ*_6_′ over the entire study period (Wieloch *et al*., 2025). These relationships are thought to derive from leaf‐level ^13^C discrimination by glyceraldehyde‐3‐phosphate dehydrogenases affecting *Δ*_4_′ and enzymes modifying the carbon–carbon double bond of phospho*enol*pyruvate affecting *Δ*_5_′ and *Δ*_6_′ (Wieloch *et al*., 2021, 2022b).

**Table 1:** Isotope‐environment signals in *Δ*_*i*_′ and their proposed enzymatic origins (underlying *Δ*_*i*_′ models shown in Supporting Information Table S1; signal origins shown in Fig. S1).

Covariate	Relationship	Period	Proposed origin of introduction		Discrimination type
			Tissue	Enzyme	
*Δ*_1_′ ~ *ε*_met_ ^a^	Negative	83–95	Stem	PGI, G6PD	PR
*Δ*_1_′ ~ *VPD*	Negative	83–95	Leaf	Rubisco,^b^ PGI, G6PD	DR and PR
*Δ*_2_′ ~ *ε*_met_ ^a^	Negative	83–95	Stem	PGI	PR
*Δ*_3_′ ~ *VPD*	Negative	83–95	Leaf	Rubisco^b^	DR
*Δ*_4_′ ~ *RAD*	Negative	64–95	Leaf	p‐GAPDH, np‐GAPDH	PR
*Δ*_4_′ ~ *TMP*	Positive	64–95	Leaf		
*Δ*_5‐6_′ ~ *RAD*	Negative	64–95	Leaf	PEPC, PK, DAHPS, Enolase	PR
*Δ*_5‐6_′ ~ *TMP*	Positive	64–95	Leaf		

*ε*_met_ denotes hydrogen isotope fractionation by metabolic processes at glucose H^1^ and H^2^. *Δ*_*i*_′ and *Δ*_5–6_′ denote ^13^C discrimination at glucose carbon position, *i*, and the arithmetic average of *Δ*_5_′ and *Δ*_6_′, respectively. DR and PR refer to diffusion‐rubisco and post‐rubisco discrimination, respectively. Glucose was extracted across an annually resolved tree‐ring series of *Pinus nigra* from the Vienna Basin. Climate data series: *RAD*, April–September global radiation (data available from 1964); *TMP*, March–October air temperature; *VPD*, March–November air vapour pressure deficit. Enzymes: DAHPS, 3‐Deoxy‐d‐*arabino*‐heptulosonate‐7‐phosphate synthase; G6PD, glucose‐6‐phosphate dehydrogenase; np‐ and p‐GAPDH, nonphosphorylating and phosphorylating glyceraldehyde‐3‐phosphate dehydrogenase; PEPC, phospho*enol*pyruvate carboxylase; PGI, phosphoglucose isomerase; PK, pyruvate kinase.

^a^ Replacing *ε*_met_ by March–July precipitation results in models with only slightly reduced explanatory power.

^b^ ^13^C discrimination during CO_2_ diffusion and assimilation by rubisco is introduced into carbon metabolism at rubisco.

Here, the relative contributions of these intramolecular ^13^C signals to whole‐glucose ^13^C discrimination (*Δ*_glu_) were estimated by variance component analysis (Notes S2). As glucose extracted from tree rings largely derives from cellulose, the results can be expected to also apply to tree‐ring cellulose (*Δ*_trc_). They are used for a critical assessment of the classical concepts and practices of carbon‐isotope dendrochronology. Subsequently, the potential value of intramolecular ^13^C analysis for constraining impacts of tropospheric ozone on forest metabolism and productivity is discussed. Lastly, it is tested whether intramolecular ^13^C signals can also be extracted from whole‐molecule (*Δ*_glu_) data.

## Components of *Δ*_glu_ variation and implications for reconstructions of leaf intrinsic water‐use efficiency

Leaf *iWUE* is regarded as an important functional property of plant ecosystems and a key determinant in the response of biogeochemical cycles to climate change (Beer *et al*., 2009). Retrospective assessment of *iWUE* relies on *Δ*_trc_ analysis, which, in turn, relies on the assumption that DR discrimination governs *Δ*_trc_ variability (Ma *et al*., 2021; Cernusak & Ubierna, 2022; Saurer & Voelker, 2022). Here, this assumption is critically examined.

**Fig. 1:**
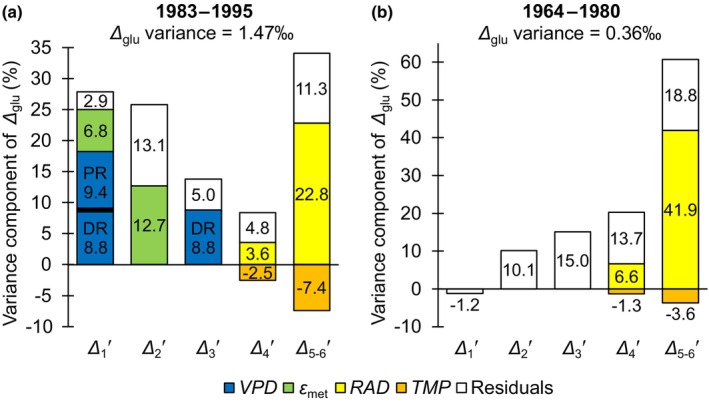
Percent contributions of intramolecular carbon‐isotope signals and model residuals to *Δ*_glu_ variation for the late (a) and early (b) period. According to current interpretation, the vapour pressure deficit (*VPD*) signal goes back to both diffusion‐rubisco (DR) and post‐rubisco (PR) discrimination (blue bars). All other signals go back to PR discrimination (green, yellow, and orange bars). Model residuals are shown as white bars. *ε*_met_ denotes hydrogen isotope fractionation by metabolic processes at glucose H^1^ and H^2^. *Δ*_*i*_′, *Δ*_5–6_′, and *Δ*_glu_ denote ^13^C discrimination at glucose carbon position, *i*, and arithmetic averages of *Δ*_5_′ and *Δ*_6_′ and the whole molecule, respectively. *RAD* and *TMP* denote April–September global radiation and March–October air temperature, respectively. Glucose was extracted across an annually resolved tree‐ring series of *Pinus nigra* from the Vienna Basin.

1. Fig. 1(a) shows percent contributions of intramolecular isotope signals found by modelling and model residuals to *Δ*_glu_ variation for the more dynamic late period (*Δ*_glu_ variance = 1.47‰) (Wieloch *et al*., 2025). Leaf ^13^C discrimination accounts for *c*. 43.5% of the total *Δ*_glu_ variance while stem ^13^C discrimination (related to *ε*_met_) accounts for *c*. 19.5%. The rest is residual variance (Notes S3). PR discrimination at the leaf‐ and stem‐level (*c*. 25.9% and 19.5%, respectively) each exceed the contribution of DR discrimination (*c*. 8.8% × 2 = 17.6%).
2. Similarly, Fig. 1(b) shows percent contributions of intramolecular isotope signals found by modelling and model residuals to *Δ*_glu_ variation for the less dynamic early period (*Δ*_glu_ variance = 0.36‰). Evidently, the contribution of *Δ*_1_′ to *Δ*_glu_ is negligible. Moreover, measurement error can account for the entire variation in *Δ*_2_′ (Notes S3). Hence, *Δ*_1_′ and *Δ*_2_′ are not considered further. However, *c*. 50% of the total *Δ*_3_′ variance may be systematic unmodelled variance (Notes S3). If we assume this variation results from DR discrimination, then DR discrimination accounts for *c*. 7.5% of the total *Δ*_glu_ variance (*c*. 0.5 × 15%) while PR discrimination accounts for *c*. 43.6%.

Hence, during both periods, DR discrimination is a comparably small contribution to total *Δ*_glu_ variation, which argues against using *Δ*_trc_ for reconstructions of interannual *iWUE* variation. As the *iWUE* signal is better resolved at the intramolecular level, *Δ*_*i*_′ analysis is expected to yield better estimates of *iWUE*.

## Physiological interpretation of climate signals in *Δ*_trc_

Currently, all reported *Δ*_trc_–climate relationships are interpreted with respect to DR discrimination (Battipaglia & Cherubini, 2022; Churakova *et al*., 2022; Gagen *et al*., 2022; van der Sleen *et al*., 2022). Thus, consideration is given only to two initial steps in the biosynthesis of tree‐ring cellulose whereas ^13^C discrimination by the numerous reactions downstream of rubisco (PR discrimination) is assumed to be constant (Fig. S1). However, recent reports of multiple intramolecular isotope signals in tree‐ring glucose (Table 1) call for a critical reassessment of this practice.

At the site discussed here, DR discrimination responds to *VPD* (for information about the site, see notes S1 in Wieloch *et al*., 2025). However, while DR discrimination accounts for *c*. 17.6% of the total variation of *Δ*_glu_ during the late period, *VPD*‐dependent PR discrimination accounts for an additional *c*. 9.4% (Fig. 1a). Hence, both DR and PR discrimination contribute to the *VPD* signal in *Δ*_glu_ and their combined contribution accounts for *c*. 27% of the total *Δ*_glu_ variance. Interestingly, simple linear regression between *Δ*_glu_ and *VPD* falsely suggests that *VPD* accounts for *c*. 54% of the total *Δ*_glu_ variance (Fig. S2). This twofold overestimation of the actual *VPD* signal likely results from intercorrelation of *VPD* with other climate parameters that also affect *Δ*_glu_. For instance, *RAD* affects tree‐ring glucose C‐5 and C‐6 (Fig. 1a), and there is significant intercorrelation between *RAD* and *VPD* (*r* = 0.6, *P* < 0.05, *n* = 13) which will result in overestimation of the *VPD* signal in *VPD*‐*Δ*_glu_ simple linear regression.

More importantly, relationships of *Δ*_glu_ with *RAD* and *TMP* derive from leaf‐level PR discrimination (Wieloch *et al*., 2021, 2022b, 2025), and *RAD*‐dependent PR discrimination alone exceeds the contribution of DR discrimination to *Δ*_glu_ variation (Fig. 1, early period, *c*. 48.5% vs *c*. 7.5%; late period, *c*. 26.4% vs *c*. 17.6%). Similarly, relationships of *Δ*_glu_ with *ε*_met_ and *PRE* derive from stem‐level PR discrimination (Wieloch *et al*., 2025), and *ε*_met_‐dependent PR discrimination contributes similarly to *Δ*_glu_ variation as DR discrimination (Fig. 1a; *c*. 19.5% and 17.6%, respectively).

Hence, *RAD*‐, *TMP*‐, *PRE*‐, and a fraction of the *VPD*‐dependent *Δ*_glu_ variation is not caused by DR discrimination and associated physiological processes. Instead, most of the climate information in *Δ*_glu_ derives from PR discrimination and associated physiological processes.

## New information from old archives – the impact of tropospheric ozone on forest metabolism

As shown recently, tree‐ring glucose carries numerous carbon (and hydrogen) isotope signals (Wieloch *et al*., 2018, 2022a), and there is considerable interest as to their scientific value in plant ecophysiology and biogeochemistry. For instance, the *RAD*‐dependent carbon‐isotope signal at tree‐ring glucose C‐5 and C‐6 (Table 1) is thought to originate from ozone‐induced metabolic adjustments (Wieloch *et al*., 2022b). *RAD* promotes the photochemical formation of tropospheric ozone (Ainsworth *et al*., 2012) which causes downregulation of rubisco and upregulation of PEPC (Saurer *et al*., 1995; Dizengremel, 2001). Additionally, 3‐deoxy‐d‐*arabino*‐heptulosonate‐7‐phosphate synthase is expressed (Janzik *et al*., 2005; Betz *et al*., 2009). These biochemical adjustments can be expected to result in increased relative carbon flux into mitochondrial metabolism and the shikimate pathway (Fig. S1; Dizengremel, 2001). Hence, the isotope signal at C‐5 and C‐6 can potentially be used to reconstruct tropospheric ozone concentration, and ozone effects on forest metabolism and productivity.

In 2100, ozone is predicted to cause forest productivity losses of 17% relative to preindustrial air, which would have severe adverse effects on global carbon cycling and climate change (Wittig *et al*., 2009). However, this estimate relies strongly on short‐term experiments on tree seedlings and saplings and may therefore not apply to mature natural forests (Emberson, 2020). The tree‐ring isotope signal at glucose C‐5 and C‐6, on the other hand, can potentially be used to constrain ozone effects on mature natural forests across space and time. Similarly, other intramolecular carbon and hydrogen isotope signals detected in tree‐ring glucose may help to advance our knowledge about other aspects of forest metabolism (Wieloch *et al*., 2025).

## Mining whole‐molecule data for information seen at the intramolecular level

Over the past decades, dendrochronologists have collected a wealth of (whole‐molecule) *Δ*_trc_ data covering various forest biomes world‐wide (e.g. Battipaglia & Cherubini, 2022; Churakova *et al*., 2022; van der Sleen *et al*., 2022). These data (*per se*) contain the same valuable information as (intramolecular) *Δ*_*i*_′ data. However, since *Δ*_trc_ has sixfold lower resolution than *Δ*_*i*_′, clear‐cut extraction of *Δ*_*i*_′‐environment signals from *Δ*_trc_ data may not be feasible.

To test this, *Δ*_glu_ data of both study periods were modelled as function of all covariates known to significantly affect *Δ*_*i*_′ (cf Tables 1, S1). It was found that, during the late period, *Δ*_glu_ is significantly related to *ε*_met_ and *RAD* (Table 2, *P* ≤ 0.01, *n* = 13), and close to significantly related to *VPD* and *TMP* (*P* ≤ 0.15). By increasing the number of observations, all relationships might become significant. Moreover, the slope estimates of the *Δ*_glu_ model are not significantly different from those of the *Δ*_*i*_′ models (Fig. S3). During the early period, *Δ*_glu_ is significantly related to *RAD* (Table 2, *P* ≤ 0.005, *n* = 15) but not *TMP* (*P* = 0.39). Still, the slope estimates of the *Δ*_glu_ model are not significantly different from those of the *Δ*_*i*_′ model (Fig. S4). Lastly, the change point separating the two study periods is detectable at both the intramolecular (*Δ*_1–3_′) and whole‐molecule (*Δ*_glu_) level (Wieloch *et al*., 2025).

**Table 2:** Multiple linear regression models of *Δ*_glu_ as function of *ε*_met_, *VPD*, *RAD*, and *TMP*.

*Δ*_glu_ ~ *ε*_met_ + *VPD* + *RAD* + *TMP*, 1983–1995			
*R* ^2^ = 0.86, adj*R* ^2^ = 0.79, *P* < 0.002, *n* = 13			
	Estimate	±SE	*P*
Intercept	25.0	5.2	≤ 0.001
*ε*_met_	−0.0142	0.0042	≤ 0.01
*VPD*	−0.00753	0.00475	= 0.15
*RAD*	−0.00475	0.00142	≤ 0.01
*TMP*	0.686	0.411=	= 0.13

*Δ*_glu_ ~ *RAD* + *TMP*, 1964–1980			
*R* ^2^ = 0.5, adj*R* ^2^ = 0.42, *P* = 0.015, *n* = 15			
	Estimate	±SE	*P*
Intercept	21.2	3.8	≤ 0.0001
*RAD*	−0.00350	0.00102	≤ 0.005
*TMP*	0.242	0.269	= 0.39

*Δ*_glu_ and *ε*_met_ denote whole‐molecule ^13^C discrimination of glucose and average hydrogen isotope fractionation caused by metabolic processes at glucose H^1^ and H^2^, respectively. Glucose was extracted across an annually resolved tree‐ring series of *Pinus nigra* from the Vienna Basin. Climate data series: *RAD*, April–September global radiation; *TMP*, March–October air temperature; *VPD*, March–November air vapour pressure deficit.

Taken together, in the present case, most of the isotope‐environment signals evident in *Δ*_*i*_′ can also be extracted from *Δ*_glu_. Hence, reanalyses of existing *Δ*_trc_ datasets based on recent insights into plant isotope fractionation may yield both more accurate estimates of ecophysiological properties linked to DR discrimination (such as *iWUE*) and novel information about ecophysiological properties linked to PR discrimination (such as metabolic responses to ozone). That said, in *Δ*_trc_ analysis, the intramolecular location of any isotope‐environment signal will always remain unknown which adds a level of uncertainty regarding the signal's metabolic origin and process specificity.

## Conclusions and outlook

The picture emerging here is inconsistent with the classical (DR discrimination‐centred) concepts and practices of carbon‐isotope dendrochronology. Evidently, processes downstream of rubisco in leaves and stems introduced most of the isotope signals and variation in the tree‐ring series examined. Hence, most of the ecophysiological and climate information in this record relates to PR processes. This opens new and exciting research avenues. First, the isotope signal reflecting *iWUE* is better resolved at the intramolecular than at the whole‐molecule level. Careful separation of this signal from other signals in *Δ*_*i*_′ or *Δ*_trc_ is expected to yield more accurate estimates of *iWUE*. Second, an isotope signal at tree‐ring glucose C‐5 and C‐6 reports metabolic changes in response to tropospheric ozone. Ozone is known for its severe adverse effects on forest productivity, global carbon cycling, and climate change. Analysing the signal at C‐5 and C‐6 may help to constrain these effects in natural forest ecosystems. Third, *Δ*_*i*_′ analysis gives access to deconvoluted information about multiple climate parameters and is therefore expected to enable distinctly more comprehensive paleoclimate reconstructions than *Δ*_trc_ analysis, providing an improved baseline for climate predictions (Wieloch *et al*., 2025). Fourth, recent and future insights into plant ^13^C discrimination from *Δ*_*i*_′ analysis may enable extraction of information about multiple ecophysiological processes from existing *Δ*_trc_ datasets.

Taken together, *Δ*_*i*_′ analysis has significant disruptive potentials regarding the scientific development of the field of carbon‐isotope dendrochronology. Unfortunately, measuring *Δ*_*i*_′ by nuclear magnetic resonance spectroscopy is labour‐intensive and requires technology and know‐how inaccessible to most dendrochronological laboratories. However, protocols enabling *Δ*_*i*_′ measurements by Orbitrap mass spectrometry are currently under development and may soon make *Δ*_*i*_′ data broadly accessible (Dion‐Kirschner *et al*., 2023; Neubauer *et al*., 2023; Gessler *et al*., 2024). Moving from whole‐molecule to intramolecular tree‐ring isotope analysis is comparable to using a more powerful microscope and promises novel information about metabolism and climate across space and time (Wieloch *et al*., 2018, 2021, 2022a,b, 2025; Gessler *et al*., 2024).

## Competing interests

None declared.

## Supporting information


**Notes S1** Isotope data.
**Notes S2** Variance component analysis.
**Notes S3** Model residuals.
**Fig. S1** Proposed metabolic origins of carbon‐isotope signals in tree‐ring glucose.
**Fig. S2** Linear regression between whole‐molecule ^13^C discrimination of tree‐ring glucose and March–November air vapour pressure deficit for the late period.
**Fig. S3** Comparison of slope estimates from the whole‐molecule vs intramolecular isotope‐environment models for the late study period.
**Fig. S4** Comparison of slope estimates from the whole‐molecule vs intramolecular isotope‐environment models for the early study period.
**Table S1** Multiple linear regression models of *Δ*_*i*_′ as function of *ε*_met_, March–November air vapour pressure deficit, March–July precipitation, April–September global radiation, and March–October air temperature.
Please note: Wiley is not responsible for the content or functionality of any Supporting Information supplied by the authors. Any queries (other than missing material) should be directed to the *New Phytologist* Central Office.

## Data Availability

The author declares that the data supporting the findings of this study are available within the paper (Fig. 1; Tables 1, 2) and its Supporting Information (Notes S3; Figs S2–S4; Table S1).
