# Supplementary material for: Shining a new light on the classical concepts of carbon‐isotope dendrochronology
Source: New Phytol. 2024 Nov 19;245(3):939–44. doi: 10.1111/nph.20258 (PMC11711932; doi:10.1111/nph.20258)
Supplement: Supplementary file 1 — Notes S1 Isotope data. Notes S2 Variance component analysis. Notes S3 Model residuals. Fig. S1 Proposed metabolic origins of carbon‐isotope signals in tree‐ring glucose. Fig. S2 Linear regression between whole‐molecule 13C discrimination of tree‐ring glucose and March–November air vapour pressure deficit for the late period. Fig. S3 Comparison of slope estimates from the whole‐molecule vs intramolecular isotope‐environment models for the late study period. Fig. S4 Comparison of slope estimates from the whole‐molecule vs intramolecular isotope‐environment models for the early study period. Table S1 Multiple linear regression models of Δ i ′ as function of ε met, March–November air vapour pressure deficit, March–July precipitation, April–September global radiation, and March–October air temperature. Please note: Wiley is not responsible for the content or functionality of any Supporting Information supplied by the authors. Any queries (other than missing material) should be directed to the New Phytologist Central Office. [file NPH-245-939-s001.pdf]

New Phytologist Supporting Information

Article title: **Shining a new light on the classical concepts of carbon-isotope dendrochronology**

Authors: Thomas Wieloch

Article acceptance date: 22 October 2024

The following Supporting Information is available for this article:

Notes S1 Isotope data.

Notes S2 Variance component analysis.

Notes S3 Model residuals.

Figure S1 Proposed metabolic origins of carbon isotope signals in tree-ring glucose.

Figure S2 Linear regression between whole-molecule ^{13}C discrimination of tree-ring glucose and March to November air vapour pressure deficit for the late period.

Figure S3 Comparison of slope estimates from the whole-molecule versus intramolecular isotope-environment models for the late study period.

Figure S4 Comparison of slope estimates from the whole-molecule versus intramolecular isotope-environment models for the early study period.

Table S1 Multiple linear regression models of Δ_i' as function of ε_{met}, March to November air vapour pressure deficit, March to July precipitation, April to September global radiation, and March to October air temperature.

Notes S1 Isotope data.

Previously, we measured intramolecular and whole-molecule ^{13}C discrimination of glucose (Δ_i' and Δ_{glu}, respectively) across an annually resolved series of *Pinus nigra* tree rings from the Vienna Basin (Wieloch *et al.*, 2018). The dataset covers the period 1961 to 1995 but lacks measurements for 1977, 1978, 1981, and 1982 (n = 31*6). Intramolecular ^{13}C discrimination was corrected for carbon redistribution by heterotrophic triose-phosphate cycling (indicated by prime) (Wieloch *et al.*, 2018). Heterotrophic triose-phosphate cycling occurs in tree-ring cells (Hill *et al.*, 1995; Wieloch *et al.*, 2022). In simplified terms, it includes (i) the breakdown of sucrose into hexose phosphates, (ii) partial breakdown of the hexose-phosphate pool into the triose phosphates glyceraldehyde 3-phosphate and dihydroxyacetone phosphate, (iii) equilibration of the two triose-phosphate species, and (iv) hexose-phosphate resynthesis from the two equilibrated triose-phosphate species. Carbon isotope signals that were initially confined to hexose-phosphate C-1, are partially present at C-6 after triose-phosphate cycling and *vice versa*. The same applies to C-2 and C-5, and C-3 and C-4. Correcting for heterotrophic triose-phosphate cycling restores the initial intramolecular ^{13}C signal distribution. Evidence supporting the validity of the correction method was published previously (Wieloch *et al.*, 2018).

Notes 2 Variance component analysis.

Carbon isotope signals and model residuals discussed here are based on previously published multiple regression models of Δ_i' as function of environmental and physiological parameters (Wieloch *et al.*, 2024): *TMP*, March to October air temperature; *RAD*, April to September global radiation (data available from 1964); *VPD*, March to November air vapour pressure deficit; ε_{met}, metabolic hydrogen isotope fractionation at glucose H^1 and H^2. These models are shown in Table S1 and exhibit the following structure:

$$\Delta_i' = \alpha + \beta_1 VPD + \beta_2 \varepsilon_{met} + \beta_3 RAD + \beta_4 TMP + e \quad \text{(S1)}$$

where α, β_j, and e denote the model intercept, coefficients, and error, respectively. Based on these models, each Δ_i' series was deconvoluted into its variable components yielding Δ_i'~*VPD*, Δ_i'~ε_{met}, Δ_i'~*RAD*, Δ_i'~*TMP*, and Δ_i'~e series. Subsequently, the percent contribution of each component to Δ_{glu} variability was calculated as

$$C_{\Delta_i' \sim x} = \frac{\mathrm{Cov}(\Delta_i' \sim x, \Delta_{\mathrm{glu}})/6}{\mathrm{Var}(\Delta_{\mathrm{glu}})} * 100 \quad \text{(S2)}$$

where Cov(Δ_i'~x, Δ_{glu}) denotes the covariance between Δ_{glu} and a selected component where x = {*VPD*, ε_{met}, *RAD*, *TMP*, e}, and Var(Δ_{glu}) denotes the variance of Δ_{glu}.

Notes S3 Model residuals.

On average, Δ_i' data of the late period (1983 to 1995, n = 13) exhibit a standard error of ±0.86‰. Hence, not all of the variation in the Δ_i' series can be captured by modelling. Based on published procedures (Nilsson *et al.*, 1996), the variance in Δ_i' series caused by measurement error was estimated and compared with the variance of Δ_i' model residuals (Table A). For Δ_1', Δ_3', Δ_4', and $\Delta_{5\text{-}6}$', the estimated error variance is similar to the models' residual variance indicating that the former largely accounts for the latter. By contrast, measurement errors account for only *c.* 26% (0.73/2.76) of the residual variance of the Δ_2' model. Hence, this model may require extensions to capture the entire systematic Δ_2' variation. The systematic variation in Δ_2' not captured by modelling accounts for *c.* 10% of the total Δ_{glu} variation (Fig. 1A, 13.1*0.74). However, in contrast to Δ_1' and Δ_3', Δ_2' is not related to *VPD* (Wieloch *et al.*, 2024). Therefore, the systematic variation in Δ_2' likely reflects PR and not DR discrimination and can be disregarded here without affecting the main conclusions.

For the late period, Δ_1', Δ_2', and Δ_3' were modelled as function of ε_{met} and/or *VPD* (Table S1). These models do not work for the early period (Wieloch *et al.*, 2024). By contrast, *RAD* and *TMP* were found to affect Δ_4' to Δ_6' over the entire study period. On average, Δ_i' data of the early period (1964 to 1980, n = 15) exhibit a standard error of ±0.94‰. Based on published procedures (Nilsson *et al.*, 1996), the variance in Δ_i' series caused by measurement error was estimated and compared with the variance of Δ_i' model residuals (Table B). Since Δ_1', Δ_2', and Δ_3' data of the early period were not modelled, their entire variance constitutes residual variance. For Δ_2', Δ_4', and $\Delta_{5\text{-}6}$', the estimated error variance is similar to the residual variance indicating that the former largely accounts for the latter. By contrast, the residual variance of Δ_1' and Δ_3' is about 2 times larger than the estimated error variance. Hence, Δ_1' and Δ_3' may contain systematic variance which could, in principle, be captured by modelling.

Table A Estimated error variance in Δ_i' series and residual variance of Δ_i' models for the late period (1983 to 1995, n = 13).

	Δ_1'	Δ_2'	Δ_3'	Δ_4'	$\Delta_{5\text{-}6}$'
**Estimated error variance [‰]**	0.71	0.73	0.71	0.77	0.47
**Residual variance [‰]**	0.78	2.67	0.57	1.00	0.35

Table B Estimated error variance in Δ_i' series and residual variance of Δ_i' for the early period (1964 to 1980, n = 15).

	Δ_1'	Δ_2'	Δ_3'	Δ_4'	$\Delta_{5\text{-}6}$'
**Estimated error variance [‰]**	1.05	0.77	0.98	0.92	0.50
**Residual variance [‰]**	2.22	1.12	2.03	1.17	0.57

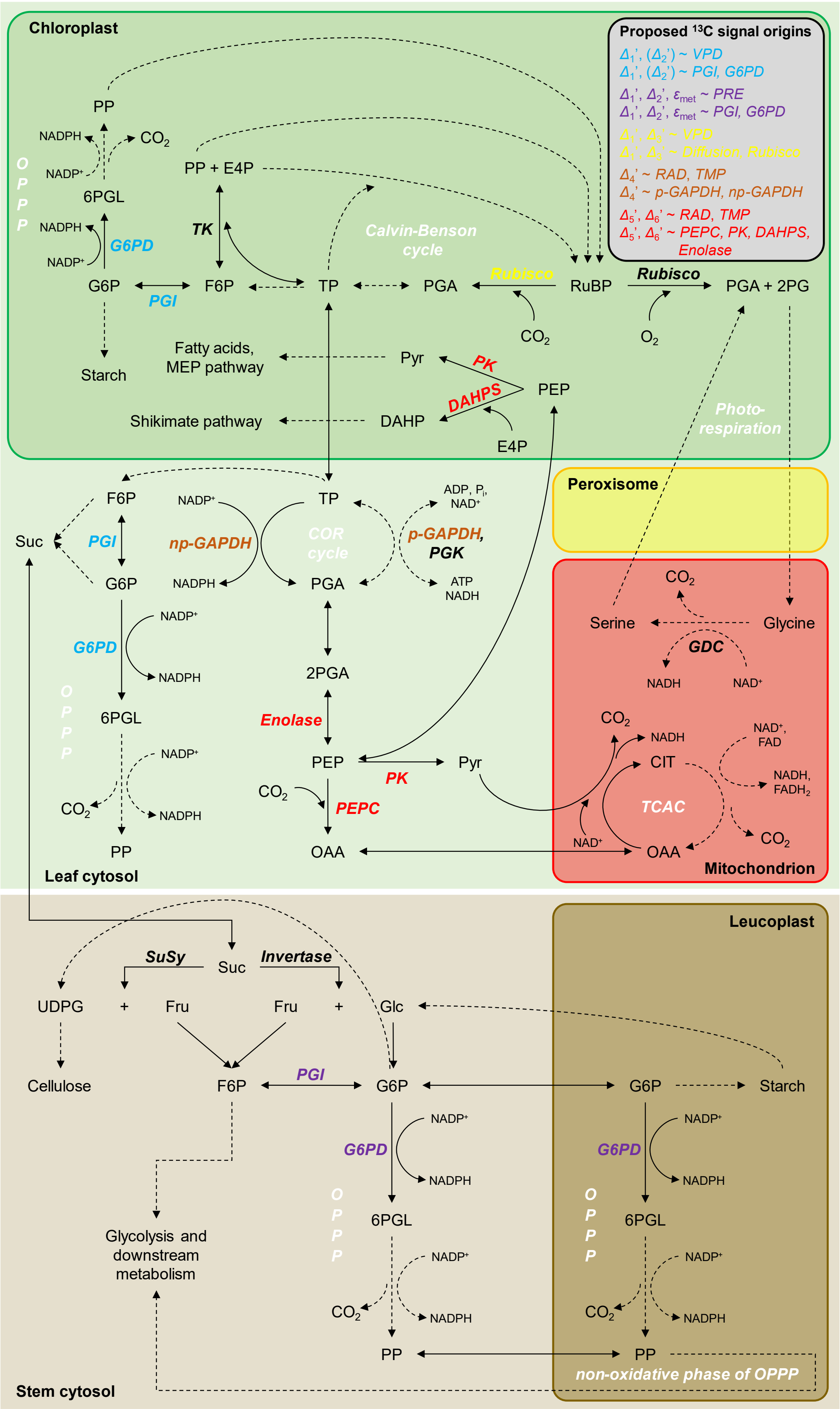
Chloroplast
Proposed 13C signal origins
Δ1', (Δ2') ~ VPD
Δ1', (Δ2') ~ PGI, G6PD
Δ1', Δ2', εmet ~ PRE
Δ1', Δ2', εmet ~ PGI, G6PD
Δ1', Δ3' ~ VPD
Δ1', Δ3' ~ Diffusion, Rubisco
Δ4' ~ RAD, TMP
Δ4' ~ p-GAPDH, np-GAPDH
Δ5', Δ6' ~ RAD, TMP
Δ5', Δ6' ~ PEPC, PK, DAHPS, Enolase
PP
NADPH
NADP+
CO2
OPPP
6PGL
G6PD
G6P
PGI
F6P
TK
PP + E4P
TP
Calvin-Benson cycle
PGA
Rubisco
RuBP
PGA + 2PG
O2
Starch
Fatty acids, MEP pathway
Pyr
PK
DAHPS
PEP
Shikimate pathway
DAHP
E4P
Photo-respiration
Peroxisome
Suc
np-GAPDH
COR cycle
p-GAPDH, PGK
ADP, Pi, NAD+
ATP
NADH
2PGA
Enolase
PEPC
OAA
Leaf cytosol
Serine
Glycine
GDC
NAD+
NAD+, FAD
NADH, FADH2
CIT
TCAC
Mitochondrion
Leucoplast
SuSy
Invertase
UDPG
Fru
Glc
Cellulose
Glycolysis and downstream metabolism
non-oxidative phase of OPPP
Stem cytosol

Figure S1 Proposed metabolic origins of carbon isotope signals in tree-ring glucose. Copied figure from a previously published article (Wieloch *et al.*, 2024). Dashed arrows indicate that intermediate reactions are not shown. Abbreviations: 2PG, 2-phosphoglycolate; 2PGA, 2-phosphoglycerate; 6PGL, 6-phosphogluconolactone; ADP, adenosine diphosphate; ATP, adenosine triphosphate; CIT, citrate; COR cycle, cytosolic oxidation-reduction cycle; DAHP, 3-Deoxy-D-*arabino*-heptulosonate 7-phosphate; DAHPS, DAHP synthase; E4P, erythrose 4-phosphate; F6P, fructose 6-phosphate; FAD, flavin adenine dinucleotide; Fru, fructose; G6P, glucose 6-phosphate; G6PD, G6P dehydrogenase; GDC, glycine decarboxylase complex; Glc, glucose; MEP pathway, methylerythritol 4-phosphate pathway; NAD^+, nicotinamide adenine dinucleotide; $NADP^+$, nicotinamide adenine dinucleotide phosphate; np-GAPDH, non-phosphorylating glyceraldehyde-3-phosphate dehydrogenase; OAA, oxaloacetate; OPPP, oxidative pentose phosphate pathway; PEP, phospho*enol*pyruvate; PEPC, PEP carboxylase; p-GAPDH, phosphorylating glyceraldehyde-3-phosphate dehydrogenase; PGA, 3-phosphoglycerate; PGI, phosphoglucose isomerase; PGK, phosphoglycerate kinase; P_i, inorganic phosphate; PK, pyruvate kinase; PP, pentose phosphate; *PRE*, precipitation; Pyr, pyruvate; Rubisco, ribulose-1,5-bisphosphate carboxylase/oxygenase; RuBP, ribulose 1,5-bisphosphate; *RAD*, global radiation; Suc, sucrose; SuSy, sucrose synthase; TCAC, tricarboxylic acid cycle; TK, transketolase; *TMP*, air temperature; TP, triose phosphates (glyceraldehyde 3-phosphate, dihydroxyacetone phosphate); UDPG, uridine diphosphate glucose; *VPD*, air vapour pressure deficit; Δ_i', intramolecular ^{13}C discrimination where *i* denotes individual glucose carbon positions and the prime denotes data corrected for ^{13}C signal redistribution by heterotrophic triose phosphate cycling; ε_{met}, metabolic deuterium fractionation at glucose H^1 and H^2.

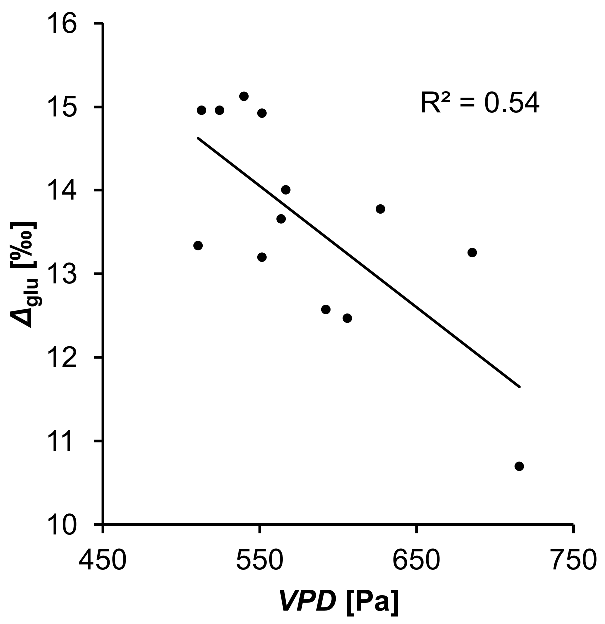

Figure S2 Linear regression between whole-molecule ^{13}C discrimination of tree-ring glucose (Δ_{glu}) and March to November air vapour pressure deficit (*VPD*) for the late period (solid line, *n* = 13). Glucose was extracted across an annually resolved tree-ring series of *Pinus nigra* from the Vienna Basin.

$$\varDelta_{\text{glu}} = \text{a}_0 + \text{a}_1\varepsilon_{\text{met}} + \text{a}_2 VPD + \text{a}_3 RAD + \text{a}_4 TMP + E_{\text{glu}}$$

$$\varDelta_1{}' = \text{b}_0 + \text{b}_1\varepsilon_{\text{met}} + \text{b}_2 VPD + E_1$$

$$\varDelta_2{}' = \text{c}_0 + \text{c}_1\varepsilon_{\text{met}} + E_2$$

$$\varDelta_3{}' = \text{d}_0 + \text{d}_1 VPD + E_3$$

$$\varDelta_{4-6}{}' = \text{e}_0 + \text{e}_1 RAD + \text{e}_2 TMP + E_{4-6}$$

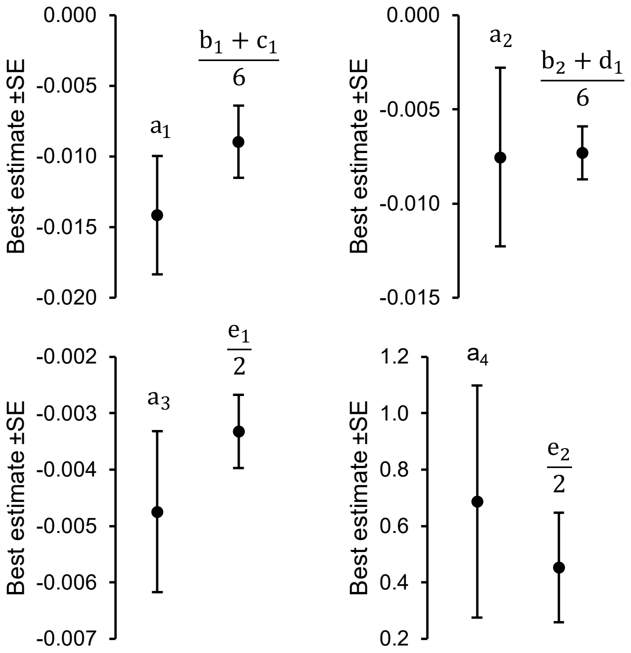

Figure S3 Comparison of slope estimates from the whole-molecule ($\varDelta_{glu}$) versus intramolecular ($\varDelta_i$') isotope-environment models for the late study period (1983 to 1995). The top part of the figure shows the structures of the linear regression models while the bottom part shows the slope comparison. Numerical details of the $\varDelta_{glu}$ and $\varDelta_i$' models are given in Tables S1 and 2, respectively. At the whole-molecule level (in $\varDelta_{glu}$), the slope of an isotope-environment relationship that is restricted to only one out of the six glucose carbon position will appear six-fold diluted. Hence, to facilitate slope comparison, all intramolecular slope estimates were scaled down to whole-molecule values. This scaling is reflected by the denominators in the bottom part of the figure. $\varDelta_i$', $\varDelta_{4\text{-}6}$', and $\varDelta_{glu}$ denote ^{13}C discrimination at glucose carbon position, *i*, and the arithmetic averages of $\varDelta_4$' to $\varDelta_6$' and the whole molecule, respectively. ε_{met} denotes hydrogen isotope fractionation by metabolic processes at glucose H^1 and H^2. Glucose was extracted across an annually resolved tree-ring series of *Pinus nigra* from the Vienna Basin. *VPD*, *RAD*, and *TMP* denote March to November air vapour pressure deficit, April to September global radiation, and March to October air temperature, respectively. *E* denotes model residuals. Whole-molecule and intramolecular slope estimates shown in each graph are not significantly different.

$$\Delta_{\text{glu}} = \text{a}_0 + \text{a}_1 RAD + \text{a}_2 TMP + E_{\text{glu}}$$

$$\Delta_{4-6}{}' = \text{b}_0 + \text{b}_1 RAD + \text{b}_2 TMP + E_{4-6}$$

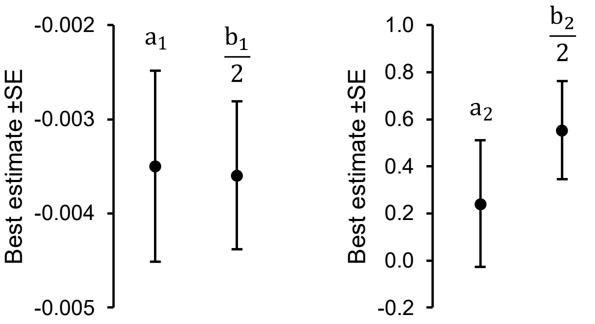

Figure S4 Comparison of slope estimates from the whole-molecule (Δ_{glu}) versus intramolecular (Δ_i') isotope-environment models for the early study period (1964 to 1980). The top part of the figure shows the structures of the linear regression models while the bottom part shows the slope comparison. Numerical details of the Δ_{glu} and Δ_i' models are given in Tables S1 and 2, respectively. At the whole-molecule level (in Δ_{glu}), the slope of an isotope-environment relationship that is restricted to three out of the six glucose carbon position will appear two-fold diluted. Hence, to facilitate slope comparison, all intramolecular slope estimates were scaled down to whole-molecule values. This scaling is reflected by the denominators in the bottom part of the figure. $\Delta_{4\text{-}6}$' and Δ_{glu} denote average ^{13}C discrimination of glucose C-4 to C-6 and the whole molecule, respectively. Glucose was extracted across an annually resolved tree-ring series of *Pinus nigra* from the Vienna Basin. *RAD* and *TMP* denote April to September global radiation (series starts in 1964) and March to October air temperature, respectively. *E* denotes model residuals. Whole-molecule and intramolecular slope estimates shown in each graph are not significantly different.

Table S1 Multiple linear regression models of Δ_i' as function of ε_{met}, March to November air vapour pressure deficit (*VPD*), March to July precipitation (*PRE*), April to September global radiation (*RAD*), and March to October air temperature (*TMP*).

**Δ_1' ~ ε_{met} + *VPD*, 1983-1995**			
R^2 = 0.87, *adj*R^2 = 0.84, p < 0.00004, n = 13			
	**Estimate**	**± SE**	***p* ≤**
**Intercept**	36.0	2.7	0.0000001
**ε_{met}**	-0.0187	0.0057	0.008
VPD	-0.0295	0.0047	0.0001
**Δ_1' ~ *PRE* + *VPD*, 1983-1995**			
R^2 = 0.82, *adj*R^2 = 0.79, p < 0.0002, n = 13			
	**Estimate**	**± SE**	***p* ≤**
**Intercept**	27.8	4.4	0.00008
PRE	0.0146	0.0061	0.04
VPD	-0.0280	0.0058	0.0007
**Δ_2' ~ ε_{met}, 1983-1995**			
R^2 = 0.54, *adj*R^2 = 0.50, p < 0.004, n = 13			
	**Estimate**	**± SE**	***p* ≤**
**Intercept**	11.8	1.7	0.00002
**ε_{met}**	-0.0351	0.0097	0.004
**Δ_2' ~ *PRE*, 1983-1995**			
R^2 = 0.43, *adj*R^2 = 0.37, p < 0.02, n = 13			
	**Estimate**	**± SE**	***p* ≤**
**Intercept**	-1.84	2.79	0.52
PRE	0.0274	0.0096	0.016
**Δ_3' ~ *VPD*, 1983-1995**			
R^2 = 0.57, *adj*R^2 = 0.53, p < 0.003, n = 13			
	**Estimate**	**± SE**	***p* ≤**
**Intercept**	14.3	2.2	0.000040
VPD	-0.0143	0.0037	0.0030
**Δ_4' ~ *RAD* + *TMP*, 1964-1995**			
R^2 = 0.15, *adj*R^2 = 0.09, p = 0.12, n = 28			
	**Estimate**	**± SE**	***p* ≤**
**Intercept**	8.33	4.73	0.09
RAD[(a)]	-0.00266	0.00160	0.1
TMP	0.931	0.439	0.04
**Δ_5' ~ *RAD* + *TMP*, 1964-1995**			
R^2 = 0.66, *adj*R^2 = 0.64, p = 0.000001, n = 28			
	**Estimate**	**± SE**	***p* ≤**
**Intercept**	24.8	4.3	0.000005
RAD	-0.0103	0.0015	0.0000002
TMP	1.81	0.40	0.0001

(Table S1 continues on the next page)

(Table S1 continued)

**Δ_6' ~ *RAD* + *TMP*, 1964-1995**			
R^2 = 0.47, $adjR^2$ = 0.43, p = 0.0003, n = 28			
	**Estimate**	**± SE**	***p* ≤**
**Intercept**	27.3	4.2	0.0000009
RAD	-0.00658	0.00144	0.0001
TMP	0.876	0.393	0.04
**$\Delta_{5\text{-}6}$' ~ *RAD* + *TMP*, 1964-1995**			
R^2 = 0.72, $adjR^2$ = 0.70, p = 0.0000001, n = 28			
	**Estimate**	**± SE**	***p* ≤**
**Intercept**	26.0	3.1	0.00000001
RAD	-0.00843	0.00105	0.00000002
TMP	1.35	0.29	0.00009
**$\Delta_{4\text{-}6}$' ~ *RAD* + *TMP*, 1964-1980**			
R^2 = 0.64, $adjR^2$ = 0.59, p = 0.002, n = 15			
	**Estimate**	**± SE**	***p* ≤**
**Intercept**	23.4	6.0	0.002
RAD	-0.00719	0.00157	0.0006
TMP	1.11	0.42	0.02
**$\Delta_{4\text{-}6}$' ~ *RAD* + *TMP*, 1983-1995**			
R^2 = 0.74, $adjR^2$ = 0.68, p = 0.001, n = 13			
	**Estimate**	**± SE**	***p* ≤**
**Intercept**	25.32	4.45	0.0002
RAD	-0.00665	0.00130	0.0004
TMP	0.907	0.388	0.04
**Δ_6' ~ *RAD* + *TMP*, 1964-1995**			
R^2 = 0.47, $adjR^2$ = 0.43, p = 0.0003, n = 28			
	**Estimate**	**± SE**	***p* ≤**
**Intercept**	27.3	4.2	0.0000009
RAD	-0.00658	0.00144	0.0001
TMP	0.876	0.393	0.04
**$\Delta_{5\text{-}6}$' ~ *RAD* + *TMP*, 1964-1995**			
R^2 = 0.72, $adjR^2$ = 0.70, p = 0.0000001, n = 28			
	**Estimate**	**± SE**	***p* ≤**
**Intercept**	26.0	3.1	0.00000001
RAD	-0.00843	0.00105	0.00000002
TMP	1.35	0.29	0.00009
**$\Delta_{4\text{-}6}$' ~ *RAD* + *TMP*, 1964-1980**			
R^2 = 0.64, $adjR^2$ = 0.59, p = 0.002, n = 15			
	**Estimate**	**± SE**	***p* ≤**
**Intercept**	23.4	6.0	0.002
RAD	-0.00719	0.00157	0.0006
TMP	1.11	0.42	0.02

(Table S1 continues on the next page)

(Table S1 continued)

$\Delta_{4\text{-}6}$' ~ ***RAD*** **+** ***TMP*****, 1983-1995**			
R^2 = 0.74, $adjR^2$ = 0.68, p = 0.001, n = 13			
	**Estimate**	**± SE**	***p* ≤**
**Intercept**	25.32	4.45	0.0002
RAD	-0.00665	0.00130	0.0004
TMP	0.907	0.388	0.04

All of these models except for the last two were published previously (Wieloch *et al.*, 2024). ε_{met}, Δ_i', $\Delta_{5\text{-}6}$', and $\Delta_{4\text{-}6}$' denote hydrogen isotope fractionation by metabolic processes at glucose H^1 and H^2, carbon isotope discrimination at glucose carbon position, *i*, the arithmetic average of Δ_5' and Δ_6', and the arithmetic average of Δ_4', Δ_5', and Δ_6', respectively. Glucose was extracted across an annually resolved tree-ring series of *Pinus nigra* from the Vienna Basin.

[a] Compared to other Δ_i' series, the systematic variance in Δ_4' is relatively low, which may explain the relatively low significance of its relationship with *RAD*.

References

Hill SA, Waterhouse JS, Field EM, Switsur VR, Ap Rees T. **1995**. Rapid recycling of triose phosphates in oak stem tissue. *Plant, Cell and Environment* **18**: 931–936.

Nilsson MB, Dåbakk E, Korsman T, Renberg I. **1996**. Quantifying relationships between near-infrared reflectance spectra of lake sediments and water chemistry. *Environmental Science & Technology* **30**: 2586–2590.

Wieloch T, Ehlers I, Yu J, Frank D, Grabner M, Gessler A, Schleucher J. **2018**. Intramolecular ^{13}C analysis of tree rings provides multiple plant ecophysiology signals covering decades. *Scientific Reports* **8**: 5048.

Wieloch T, Holloway-Phillips M, Yu J, Niittylä T. **2024**. New insights into the mechanisms of plant isotope fractionation from combined analysis of intramolecular ^{13}C and deuterium abundances in *Pinus nigra* tree-ring glucose. *New Phytologist*. https://doi.org/10.1111/nph.20113

Wieloch T, Sharkey TD, Werner RA, Schleucher J. **2022**. Intramolecular carbon isotope signals reflect metabolite allocation in plants. *Journal of Experimental Botany* **73**: 2558–2575.
